# Efficacy and Safety of Metformin Use in Rheumatoid Arthritis: A Randomized Controlled Study

**DOI:** 10.3389/fphar.2021.726490

**Published:** 2021-09-22

**Authors:** Mahmoud Gharib, Walaa Elbaz, Ebtissam Darweesh, Nagwa Ali Sabri, May Ahmed Shawki

**Affiliations:** ^1^ Pharmacy Practice and Clinical Pharmacy Department, Faculty of Pharmacy-Future University in Egypt, New Cairo, Egypt; ^2^ Department of Internal Medicine, Rheumatology, and Immunology, Faculty of Medicine-Alazhar University, Cairo, Egypt; ^3^ Clinical Pharmacy Department, Faculty of Pharmacy-Ain Shams University, Cairo, Egypt

**Keywords:** metformin, rheumatoid arthritis, DAS–28, CRP, adiponectin, quality of life

## Abstract

**Objective:** To evaluate the efficacy and safety of metformin use in rheumatoid arthritis (RA) patients receiving conventional synthetic disease modifying anti-rheumatic drugs (csDMARDs).

**Methods:** A prospective, randomized, controlled, single blinded, study was carried on 66 RA patients with moderate and high disease activity state, receiving csDMARDs. Patients were simply randomized to receive either metformin 850 mg twice daily (Metformin group, *n* = 33), or placebo twice daily (Control group, *n* = 33) in addition to their stable anti-rheumatic regimen and followed up for 6 months. Serum C-reactive protein (CRP), disease activity of 28 joints based on CRP (DAS-28-CRP), and quality of life (QOL) were evaluated at baseline and then every 3 months. Moreover, serum adiponectin was assessed at baseline and after 6 months.

**Results:** Sixty patients completed the study. Drop out was due to intolerance to metformin side effects (*n* = 3) and non-compliance (*n* = 3). Metformin significantly decreased CRP levels and DAS-28-CRP after 6 months compared to the control group (*p*-value <0.001). A significant improvement in QOL of metformin group was observed as early as after 3 months (*p*-value = 0.006) with a continued improvement observed at 6 months (*p*-value <0.001) compared to the control group. Despite the significantly higher serum adiponectin in the metformin group at baseline, it was significantly reduced after 6 months in the metformin group with median percent change of −63.49% compared to the significant increase in the control group with median percent change of 92.40%.

**Conclusion:** Metformin significantly improved inflammation, disease severity, and QOL in RA patients with high safety profile.

**Clinical Trial Registration**: Clinical-Trials.gov, identifier [NCT08363405].

## Introduction

Rheumatoid arthritis (RA) is a chronic, progressive, systemic inflammatory disease with an estimated prevalence ranging from 0.4 to 1.1% globally and 0.3% in the Egyptian population (Usenbo et al., 2015; West et al., 2018). Main risk factors for RA include genetic predisposition accounting for 60% of cases and female gender where women are two to three times more likely to develop RA compared to men (West et al., 2018). Clinical presentation of RA includes articular manifestations of pain and reduced mobility as well as extra-articular manifestations and several comorbidities related to systemic inflammation (Innala et al., 2016; West et al., 2018). All these factors contribute to poor quality of life (QOL), reduced productivity and work ability, and increased socioeconomic burdens (Matcham et al., 2014; van der Zee-Neuen et al., 2017).

Among the features of RA pathogenesis are the up-regulation of T-helper17 (Th17) differentiation and down-regulation of regulatory T (Treg) cells production shifting the synovial homeostasis towards inflammation (Lebre et al., 2008)**.** Differentiated Th17 cells secrete various inflammatory mediators such as interleukin-17A (IL-17A), interleukin-22 (IL-22), and interleukin-26 (IL-26), tumor necrosis factor-α (TNF-α) and granulocyte-macrophage colony-stimulating factor (GM-CSF) which subsequently stimulate fibroblast-like synoviocytes (FLSs) and macrophages to secrete further cytokines, and stimulate osteoclasts contributing to inflammation, synovial hyperplasia, cartilage destruction, and bone erosions (van Hamburg and Tas, 2018). Moreover, adiponectin, the most abundant adipocytokine in plasma produced mainly from white adipose tissue, has been reported to stimulate the production of many inflammatory mediators from FLS, mediating cartilage damage, and bone destruction (Choi et al., 2020)**.**

Although RA management has been improved during the last decades, many RA patients do not respond to available therapies or develops resistance to therapy over time (Smolen et al., 2016)**.** The first line management strategy of RA is based on using conventional synthetic disease modifying anti-rheumatic drugs (csDMARDs**)** (Singh et al., 2016; Smolen et al., 2020). Biologic DMARDs use has been reported to improve RA outcomes (Smolen et al., 2018)**.** However their high cost make them unaffordable for many patients and health systems (De Vera et al., 2014). Hence, alternative low-cost strategies are needed to control RA disease activity and improve patients’ QOL.

Metformin is an oral anti-diabetic agent that is widely used as first line treatment for type II diabetes (American Diabetes Associa, 2021). It has been reported to have many pleiotropic effects that are independent of its anti-hyperglycemic role including cardio-protective, anti-neoplastic, anti-aging, and anti-inflammatory effects (Wang et al., 2017; Rajaei et al., 2019)**.** Preclinical studies have shown that metformin has anti-arthritis, anti-inflammatory effects through several mechanisms including suppression of osteoclasts gene expression, down-regulation of IL-17-producing Th17 cells, up-regulation of Treg cells and lowering the production of pro-inflammatory cytokines (Kang et al., 2013; Son et al., 2014). Consistent findings have been reported in tissue cultures where metformin was shown to inhibit FLS proliferation and migration in a dose dependent fashion leading to down regulation of TNF-α, IL-1beta levels, and IL-6 gene expression (Chen et al., 2020)**.** Additionally, metformin has been shown to decrease the expression and production of adiponectin in adipocytes cell culture (Huypens et al., 2005)**.**

This study was designed to evaluate the potential benefits of metformin use as an adjuvant therapy in RA arthritis patients with moderate and high disease activity and its effect on serum adiponectin.

## Materials and Methods

### Study Design and Setting

This was a prospective, randomized, single blinded controlled study carried on 66 Egyptian RA patients. The study was conducted at Rheumatology and Immunology Unit of Internal Medicine Department, Al-Zahraa University Hospital, Cairo, Egypt.

### Ethics Consideration

The study protocol has been revised, approved by Research Ethics Committee of Experimental and Clinical Studies, Faculty of Pharmacy, Ain Shams University which is approved and registered at the Egyptian Ministry of Health (protocol approval number: 215). This study was conducted according to the 1964 Declaration of Helsinki and its later amendments. All patients were educated about the study protocol and were required to sign a written informed consent prior to participation without any obligation to complete the study. The study was registered at “Clinical-Trials.gov” with identifier number: NCT03863405.

### Patients

Adult patients (older than 18 years) were included in the study with established diagnosis of RA according to American College of Rheumatology/European league Against Rheumatism (ACR/EULAR) 2010 criteria (Aletaha et al., 2010), presented with moderate to high disease activity identified as disease activity score-28 based on C-reactive protein (CRP) levels (DAS-28-CRP) >3.2, receiving stable regimen of one or more csDMARDs for at least the past 3 months. Exclusion criteria were; a known hypersensitivity to metformin, prior diagnosis with diabetes mellitus, receiving metformin for any other indications, receiving biologic DMARDs therapy, impaired liver functions (liver transaminases level ≥ three times upper normal limits), impaired kidney functions (estimated glomerular filtration rate (eGFR) < 30 ml/min), pregnancy and lactation, as well as the presence of any of the following comorbidities including congestive heart failure, history of myocardial infarction, severe anemia, active infections, other inflammatory diseases, and malignancies.

Patients were simply randomized, using computerized random sample generator to either metformin group who received their csDMARDs in addition to metformin 850 mg twice daily (Haffner et al., 2005) for 6 months or control group who received their csDMARDs in addition to placebo twice daily for 6 months.

## Methods

At baseline, demographics and clinical characteristics were evaluated for all patients. Serum CRP levels, disease activity, and patient’s QOL were assessed at baseline and every 3 months thereafter. Disease activity was assessed using DAS-28-CRP scale which required physical examination of specific 28 joints by a blinded rheumatologist to evaluate tender joints count (TJC) and swollen joints count (SJC), serum CRP levels, and patient global health assessment (GH) of disease severity assessed on a scale from 0 to 100 mm. The activity score can be calculated according to the following formula (Castrejón et al., 2010)**:**

DAS28-CRP = 0.56* √(TJC28) + 0.28* √(SJC28) + 0.36*ln (CRP + 1) +0.014*(GH) + 0.96.

Patient’s QOL was assessed by Health Assessment Questionnaire Disability index (HAQ-DI) (Fries et al., 1980)**.** It comprises eight categories assessing the ability of patients to perform activities of daily living. Each category includes two or three questions scored from 0 (without any difficulty) to 3 (unable to do). The score of each category is the highest score among the scores of the included questions. If an aid or assistance device is used or if help is required from another individual, then the minimum score for that section is 2. The final score is calculated by summation of the scores for various categories divided by the number of categories resulting in a score from 0 to 3 where higher scores indicating poor QOL.

Assessment of serum adiponectin levels was performed at baseline and at the end of the study using commercial ELISA kits and serum samples were stored at −80°C untill analysis.

Patients were educated about the adverse effects and/or side effects of metformin and were required to report any of them. In addition, complete blood count (CBC), liver function tests and kidney function tests were routinely done every 6 weeks to evaluate the toxicity of csDMARDs.

The study primary outcomes were CRP levels and DAS-28-CRP while secondary outcomes were quality of life, serum adiponectin and metformin tolerability.

### Statistical Methods

Statistical analysis was done using IBM SPSS^®^ Statistics version 22 (IBM^®^ Corp., Armonk, NY, United States). Numerical data was expressed as mean and standard deviation or median and range as appropriate. Qualitative data was expressed as frequency and percentage. Percent change was calculated as [100*(value at 3 or 6 months–baseline value)/baseline value]. Pearson’s Chi-square test was used to examine the relation between qualitative variables. Quantitative data were tested for normality using Kolmogorov-Smirnov test and Shapiro-Wilk test. For normally distributed quantitative data, comparisons between two groups were done using Student t-test while for not normally distributed quantitative data, comparisons were done using Mann-Whitney test. Wilcoxon-Signed Rank test was used to compare two consecutive measures of non-parametric numerical variables. Friedman test (non-parametric repeated measures ANOVA) was used to compare between three consecutive measures of numerical variables followed by post-Hoc test for pair-wise comparisons. Due to multiple comparisons, *p*-value was corrected using Bonferroni method. All tests were two-tailed and *p*-value < 0.05 was considered significant.

### Sample Size Calculation

Sample size calculation was done by Statulator online calculator available at (http://statulator.com/SampleSize/ss2M.html). No previous study was available to estimate the effect size of metformin use on the disease activity of RA patients. Hence, a mean difference of 0.6 units in DAS-28 based on a previous study (van Gestel et al., 1998) and a S.D of 0.8 units were assumed. Using alpha of 0.05 and power of 80%, the minimum required sample size was estimated to be 28 in each group. Assuming 20% attrition rate, a sample size of 33 patients per group were required. At the end of the study power calculation was estimated using Power and Sample Size Calculation version 3.1.2. The study included 60 subjects: 30 experimental subjects and 30 control subjects. The true difference of DAS-28-CRP at 6 months between the two groups was 1.06 with pooled standard deviation of 0.75. Under these conditions the power was 99.9% with probability of type I error of 0.05.

## Results

### Baseline Evaluation

**FIGURE 1:**
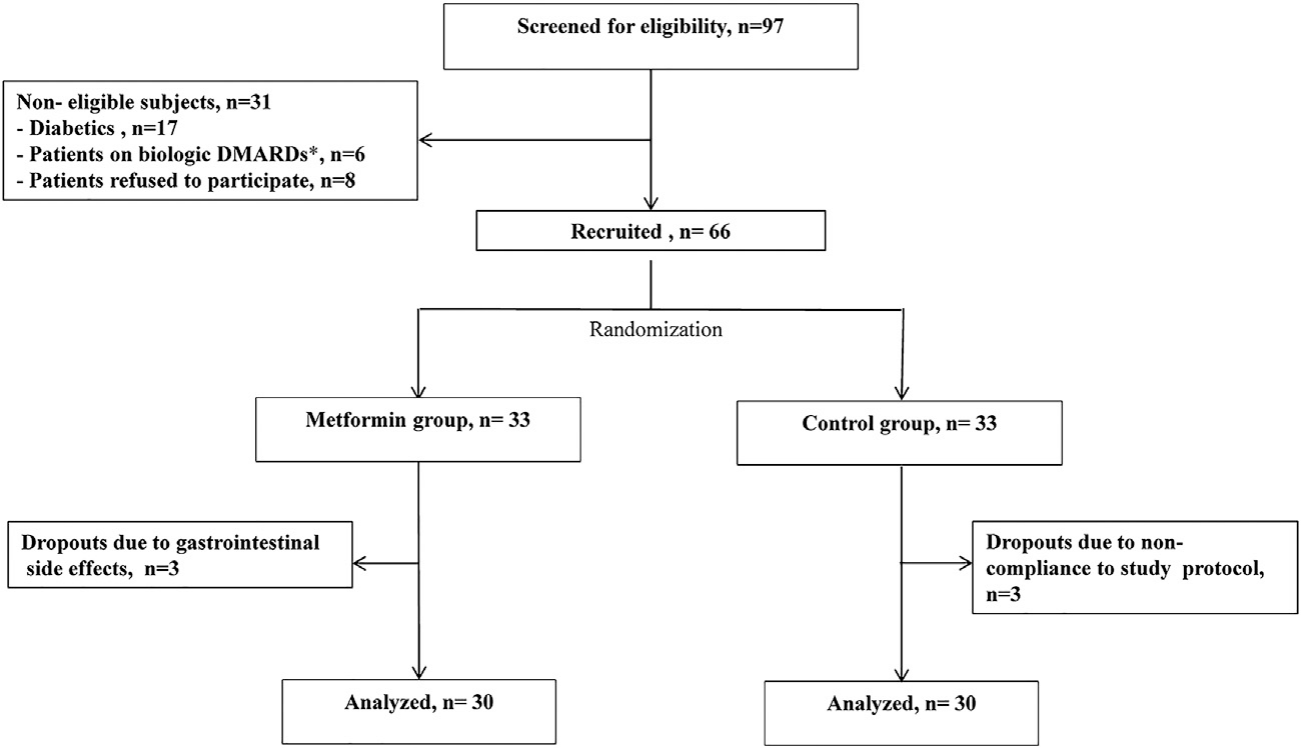
The study flow chart. *DMARDs: disease modifying anti-rheumatic drugs.

From October 2018 to March 2020, 97 patients with RA were assessed for eligibility. Only 66 patients fulfilled the inclusion criteria and were included in the study while only 60 patients completed the study. Three patients were withdrawn from the control group due to non-compliance to the study protocol while three patients left the study in the metformin group because of intolerance to gastrointestinal tract (GIT) adverse effects. The study flow chart is represented in Figure 1.

The mean age (±S.D.) of the participants was 51.1 (±8.5) years, where 43 (71.6%) of them were obese (body mass index (BMI) ≥ 30 kg/m2) and 15 (25%) were overweight (25 ≤ BMI ≤29.9). The median (range) disease duration of the study participants was 12 (4–20) years. The identified comorbidities in the study groups were hypertension, dyslipidemia, and ischemic heart disease, where 25 (41.7%) of participants had hypertension, 8 (13.3%) had dyslipidemia, and 1 (1.7%) had ischemic heart disease. The number of received DMARDS ranged from 1 to 2 with 49 (81.6%) of the participants were receiving prednisolone. There were no significant differences between groups regarding baseline demographics and clinical characteristics as shown in Table 1.

**TABLE 1:** Baseline characteristics of the study groups.

Parameter	Metformin group *n* = 30	Control group *n* = 30	*p*-value
Gender:			
Female, *n* (%)	28 (93.3)	30 (100)	^a^
Male, *n* (%)	2 (6.7)	0 (0)	
Age in years: (mean ± S.D)	(51.3 ± 9.9)	(50.9 ± 7)	0.881^b^
Weight in kg: (mean ± S.D)	(85.3 ± 12.3)	(81.5 ± 10.7)	0.207^b^
Height in m: (mean ± S.D)	(1.6 ± 0.1)	(1.6 ± 0.1)	0.630^b^
BMI in kg/m^2^: (mean ± S.D)	(32.7 ± 5.1)	(31.4 ± 3.1)	0.236^b^
Disease duration in years:			
Median (range)	13 (6–20)	12 (4–17)	0.139^c^
Comorbidities, *n* (%):			
HTN	13 (43.3)	12 (40)	0.793^d^
Dyslipidemia	4 (13.3)	4 (13.3)	1^d^
Ischemic heart disease	1 (3.3)	0 (0)	^a^
Type of csDMARDs used: *n* (%):			
LEF + SLZ	11 (36.7)	11 (36.7)	^a^
HCQ + LEF	8 (26.7)	10 (33.3)	
MTX + HCQ	6 (20)	5 (16.7)	
MTX + SLZ	3 (10)	0 (0)	
MTX + LEF	1 (3.3)	1 (3.3)	
LEF	1 (3.3)	1 (3.3)	
LEF + HCQ	0 (0)	2 (6.7)	
Corticosteroids: *n* (%):			
Not receiving steroids	5 (16.7)	6 (20)	0.943^d^
5 mg dose	5 (16.7)	5 (16.7)	
10 mg dose	20 (66.7)	19 (63.3)	

BMI: body mass index, HTN: hypertension, csDMARDs: conventional synthetic disease modifying anti-rheumatic drugs, LEF: leflunomide, SLZ: sulfasalazine, HCQ: hydroxychloroquine, MTX: methotrexate.

a No *p*-value because of small number of cases within subgroups.

b Independent t-test.

c Mann-Whitney test.

d Chi-Square test.

### Effect of Metformin on CRP Level and DAS-28-CRP

There was no significant difference between the two groups regarding serum CRP levels and DAS-28-CRP scores at baseline and after 3 months. However, after 6 months, the metformin group showed significant lower serum levels of CRP and DAS-28-CRP scores compared to the control group. These data are summarized in Table 2.

**TABLE 2:** Comparison of serum C reactive protein (CRP) levels and disease activity score based on CRP (DAS-28-CRP) between and within the study groups.

Parameter	Metformin group *n* = 30	Control group *n* = 30	*p*-value
**Serum CRP levels (mg/L)**			
Baseline, median (range)	16 (3–30)	12 (3–24)	0.204^b^
After 3 months, median (range)	12 (2–24)	14 (3–26)	0.207^b^
After 6 months, median (range)	7 (2–36)	15 (6–30)	<0.001^a^ ^,^ ^b^
*p*-value	<0.001^a^ ^,^ ^c^ ^,^ ^d^	0.117^c^	
**DAS-28-CRP**			
Baseline, median (range)	5.47 (4.32–6.87)	5.65 (3.68–6.85)	1^b^
After 3 months, median (range)	5.20 (3.36–6.66)	5.65 (3.70–6.66)	0.144^b^
After 6 months, median (range)	4.79 (3.16–6.57)	5.88 (4.3–6.8)	<0.001^a^ ^,^ ^b^
*p*-value	<0.001^a^ ^,^ ^c^ ^,^ ^e^	0.465^c^	

a Indicates statistical significance (*p*-value <0.05).

b Mann-Whitney test.

c Friedman test.

d Post-hoc tests (pairwise comparisons) revealed significance differences in CRP levels between baseline-after 3 months, baseline-after 6 months, and after 3 months-after 6 months.

e Post-hoc tests (pairwise comparisons) revealed significance differences in DAS-28 between baseline-after 3 months, baseline-after 6 months, and after 3 months-after 6 months.

Within group comparisons revealed a significant reduction of serum CRP levels after 3 and 6 months within the metformin group only, with median percent change from baseline of −26.79% and −51.67% after 3 and 6 months respectively in the metformin group compared to 8.33 and 13.49% after 3 and 6 months respectively in the control group. The same was observed for DAS-28-CRP scores where a significant reduction was detected within the metformin group after 3 and 6 months compared to baseline. The median percent changes from baseline in DAS-28-CRP were −7.63% and −16.83% after 3 and 6 months respectively in the metformin group versus 0.64 and 1.20% after 3 and 6 months respectively in the control group.

### Evaluation of patients’ QOL

At baseline, no significant difference was found between the two groups regarding QOL scores. The median percent changes in HAQ-DI were −15.79% and −20% in the metformin group compared to 0 and 6.25% in the control group after 3 and 6 months respectively. There was a significant improvement of QOL in the metformin group after 3 and 6 months compared to the baseline and compared to the control group as represented in Table 3.

**TABLE 3:** Comparison of Health Assessment Questionnaire-Disability Index (HAQ-DI) scores between and within the study groups.

HAQ-DI score	Metformin group *n* = 30	Control group *n* = 30	*p*-value
Baseline, median (range)	2.13 (0.63–2.5)	2 (1.25–2.63)	1^b^
After 3 months, median (range)	1.88 (0.38–2.38)	2.13 (1.25–2.50)	0.006^a^ ^,^ ^b^
After 6 months, median (range)	1.56 (0.38–2.25)	2.13 (1.75–2.50)	<0.001^a^ ^,^ ^b^
*p*-value	<0.001^a^ ^,^ ^c^ ^,^ ^d^	0.065^c^	

a Indicates statistical significance (*p*-value <0.05).

b Mann-Whitney test.

c Friedman test.

d Post-hoc tests (pairwise comparisons) revealed significance differences in HAQ between baseline-after 3 months, and baseline-after 6 months.

### Evaluation of Serum Adiponectin

**FIGURE 2:**
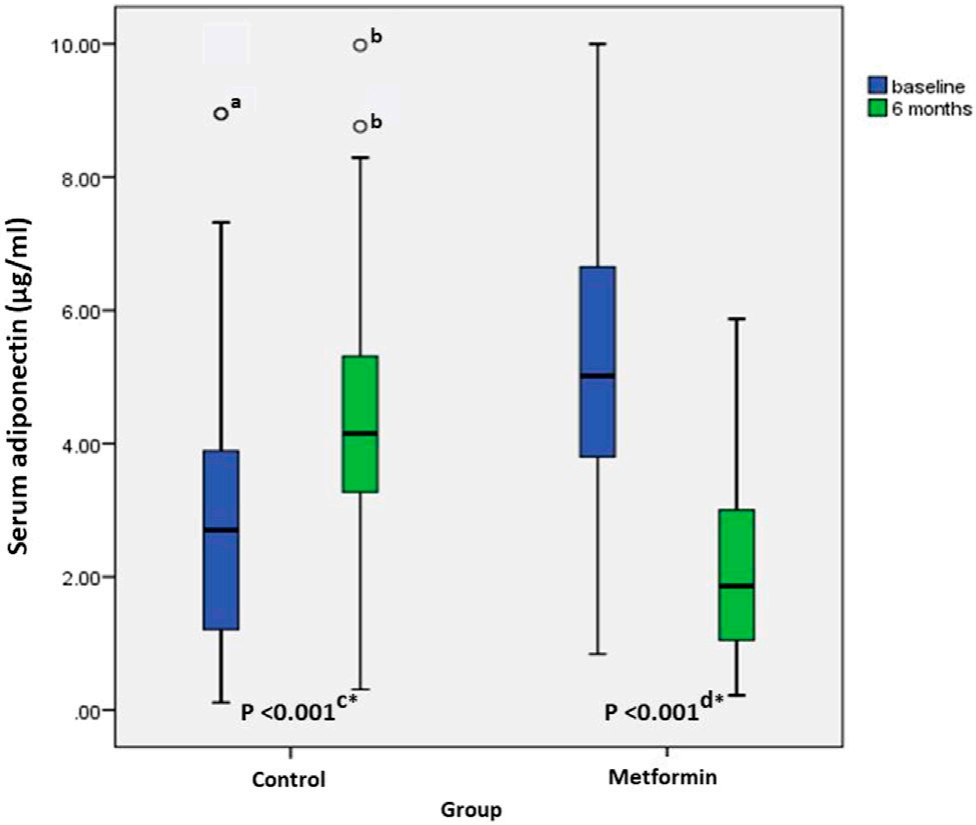
Boxplot of serum adiponectin levels of control group (*n* = 30), and metformin group (*n* = 30) at baseline, and after 6 months. Medians (ranges) of serum adiponectin levels in control group were 2.7 (0.11–8.95) and 4.15 (0.31–9.98) at baseline and after 6 months respectively compared to 5.01 (0.84–10) and 1.86 (0.22–5.87) in metformin group at baseline and after 6 months respectively. a: Outlier in control group at baseline. b: Outliers in control group after 6 months. c: Comparison between baseline and after 6 months levels of serum adiponectin in the control group using Wilcoxon Signed Rank test, d: Comparison between baseline and after 6-months levels of serum adiponectin in metformin group using Wilcoxon Signed Rank test, *: Indicates statistical significance.

At baseline, the serum adiponectin levels were significantly higher in metformin group, where the median (range) serum adiponectin levels were 5.01 (0.84–10) µg/ml compared to 2.7 (0.11–8.95) µg/ml in the control group (*p*-value <0.001). However, after 6 months the metformin group showed a significant decrease in serum adiponectin with median (range) percent change of −63.49% ([−97.80]−[−12.91]) while the control group showed significant increase with median (range) percent change of 92.40% ([−12.51]−338.24), *p*-value <0.001. Serum levels of adiponectin at baseline and after 6 months are represented in Figure 2.

### Evaluation of Tolerability of Metformin in RA Patients

Three patients in the metformin group withdrew from the study because of intolerance of metformin GIT side effects where one patient reported abdominal pain and severe diarrhea, one patient reported nausea, abdominal pain, and severe diarrhea, moreover, another one reported nausea and abdominal pain with severe flatulence. Other GIT side effects reported by the patients in both groups were mild to moderate and tolerable requiring no specific intervention and dissipated with time. These data are presented in Table 4. Routine evaluation of CBC and kidney and liver function tests did not reveal any adverse effects related to either metformin or csDMARDs use in both groups.

**TABLE 4:** Comparison of gastrointestinal side effects between the study groups.

Gastrointestinal side effects	Metformin group *n* = 30	Control group *n* = 30	*p*-value
Nausea, *n* (%)	8 (26.7)	5 (16.7)	0.347^a^
Abdominal pain ± flatulence, *n* (%)	7 (23.3)	4 (13.3)	0.317^a^
Diarrhea, *n* (%)	6 (20)	4 (13.3)	0.488^a^

a Chi-Square test.

## Discussion

Despite the availability of updated therapies for RA, many patients are poorly controlled and need intervention (Smolen et al., 2018). Drug discovery is a very complex process facing many challenges and associated with high cost where the success rate has been estimated to be only 2% (Parisi et al., 2020). Exploring the efficacy of already existing drugs in new indications is a very promising approach offering the opportunity to benefit from the already established drugs with known pharmacokinetic characteristics and safety profiles, as well as, to reduce costs and save time (Pushpakom et al., 2019)**.**

This is the first randomized controlled clinical study to evaluate the effect of metformin as an adjunctive therapy to csDMARDs on the disease activity of RA patients. This study used CRP levels and DAS-28-CRP as the primary outcomes to evaluate the efficacy of metformin. C-reactive protein is a nonspecific inflammatory marker that has been used as a tool to evaluate RA progression and treatment response and can be correlated with disease severity (Wells et al., 2009). Since this was the first study to evaluate metformin use in RA patients, the dose of metformin was determined based on its recommended dose in treatment of diabetes which ranges from 500 to 2,500 mg/day (Nathan et al., 2009) to ensure safety. A dose of 850 mg twice daily of metformin was used in this trial based on the findings of the Diabetes Prevention Program study where the same dose was reported to significantly decrease CRP levels in individuals with impaired glucose tolerance with median percent reduction of 7 and 14% in males and females respectively (Haffner et al., 2005). In accordance with the previously mentioned results, the current study has shown that metformin significantly decreased serum CRP levels in RA patients compared to control indicating that metformin has potential anti-inflammatory effect.

In addition to decreasing inflammation in RA patients, metformin also ameliorated the disease severity and improved the clinical manifestations of RA in terms of DAS-28-CRP scores. The DAS-28 is one of the recommended assessment tools by ACR and EULAR guidelines to follow up RA patients’ responses to offered treatments (Singh et al., 2016; Smolen et al., 2016). There are two versions of DAS-28; ESR based score (Prevoo et al., 1995) and CRP based score (Castrejón et al., 2010). In the current study, CRP was used in the calculation of DAS-28 because CRP has many advantages over ESR, as CRP is a direct indicator of the inflammation, and its levels change rapidly according to the changes of patients’ inflammatory status. Moreover, CRP is not affected by abnormalities in erythrocytes, and possibly its levels are not affected by age and gender to the same extent observed with ESR (Siemons et al., 2014).

There are many unmet needs for RA patients including pain, fatigue, impaired physical and mental functioning, decreased work productivity, and reduced daily living activities (Taylor et al., 2016), therefore evaluation of QOL related factors in RA patients should be taken in consideration and should be assessed independently from the medical condition (Wysocka-Skurska et al., 2016)**.** Health Assessment Questionnaire Disability index is commonly used for assessment of functional status and QOL of RA patients, having the advantages of being reliable, validated, strongly correlated with clinical and laboratory markers of inflammation, and a good predictor of the long term outcomes and mortality in RA patients (Maska et al., 2011) as well as it is available in a validated Arabic form. In the current work, HAQ-DI scores significantly improved in metformin group compared to control indicating better QOL and disease control in RA patients.

Studies have reported contradictory results regarding adiponectin roles regarding inflammation, whereas adiponectin was shown to have pro-inflammatory (Lee et al., 2014) and anti-inflammatory roles (Wang et al., 2016). A recent meta-analysis of 11 studies included 813 RA patients and 684 controls showed that, the circulating adiponectin levels have been found to be elevated in patients with RA compared to controls (Lee and Bae, 2018)**.** Moreover, in a recent Swedish study included follow-up of obese subjects, high baseline serum adiponectin levels were found to be associated with an increased risk for RA development and this association was found to be independent of CRP levels (Zhang et al., 2020). In this work, at baseline, the two groups were not comparable regarding their serum adiponectin levels, where subjects of the metformin group showed significantly higher levels compared to the control group. Adiponectin can be affected by several factors including age, BMI, degree of systemic inflammation, and dietary habits where consumption of vegetables, vegetable oils, beverages such as coffee and tea was reported to increase serum adiponectin levels (Ostrowska et al., 2013). Hence, inter-patients’ variability in these factors might have contributed to this difference at baseline. The percent change of serum adiponectin levels after 6 months compared to baseline was performed to overcome this difference. Assuming that no changes in dietary habits were reported in the current study, changes in serum adiponectin levels after metformin administration could reflect the change of inflammatory state in these patients. This study showed a significant decrease of serum adiponectin levels in the metformin group while a significant increase in the control group. Reduction of serum adiponectin levels in metformin group was in accordance with the improvement in CRP levels supporting pro-inflammatory roles of adiponectin in RA and the evidence that metformin has a potential anti-inflammatory effect.

Evaluation of safety of metformin in RA patients revealed no major safety concerns in the two groups during the entire study duration. However, GIT disturbances were the most commonly reported side effects by the patients in both groups including nausea, abdominal pain, flatulence, and diarrhea. These side effects were severe in three patients who withdrew from the study because of intolerance to metformin use. It has been reported that GIT effects associated with metformin use affect up to 25% of the users, and only 5% can’t tolerate these side effects at all (McCreight et al., 2016)**.** These side effects can be avoided or minimized by using up-titration regimen similar to that used in diabetic patients where metformin is usually initiated at 850 mg once daily and gradually up-titrated to the required maintenance dose over a period that may be up to 1 month (Nathan et al., 2009).

It is worthy to mention that metformin might have additional benefits in RA patients due to its possession of positive effects on cardiac outcomes, including reduced cardiac ischemia, myocardial infarction, cardiovascular death, and all-cause mortality in patients with type II diabetes (Griffin et al., 2017). Hence, it might decrease cardiovascular complications in RA patients which could be investigated in future studies as an additional outcome in RA patients.

This study was limited by small sample size, being a single center study, and of short duration period. In addition, low male to female ratio was observed in the current study. This could be attributed to the fact that RA is three times more prevalent in females than in males. Besides, non-adherence to the study protocol was more common in males and was the rationale behind exclusion of three males from the data analysis. Further multi-center, long term studies with larger number of patients are suggested to confirm these findings and to investigate other potential benefits of metformin in patients with RA including cardiovascular protection and possible mortality benefits.

## Conclusion

Addition of metformin to csDMARDs in RA patients significantly decreased serum CRP and adiponectin levels reflecting its potential anti-inflammatory effects. Moreover, metformin decreased the disease activity and improved patients’ QOL. Metformin has many benefits including being of low cost and high tolerability in most of patients. Consequently, metformin could be suggested as a candidate add-on therapy to csDMARDs in RA patients.

## Data Availability

The raw data supporting the conclusion of this article will be made available by the authors, without undue reservation.
